# A rare axillary artery branching variation: case report in a Brazilian cadaver

**DOI:** 10.1590/1677-5449.202500682

**Published:** 2025-11-10

**Authors:** Gabriel Vitório de Araújo Suassuna, Clara Medeiros Midena, Wigínio Gabriel de Lira Bandeira, Mauro Bezerra Montello, Bento João da Graça Azevedo Abreu, Judney Cley Cavalcante

**Affiliations:** 1 Universidade Federal do Rio Grande do Norte – UFRN, Centro de Biociências, Departamento de Morfologia, Laboratório de Anatomia Humana, Núcleo de Estudo e Pesquisa em Anatomia Humana – NEPAH, Natal, RN, Brasil.

**Keywords:** anatomical variations, axillary artery, brachial artery, human anatomy, upper limb, variações anatômicas, artéria axilar, artéria braquial, anatomia humana, membro superior

## Abstract

Knowledge of vascularization is essential for surgical planning and interventional procedures in the axillary region. The axillary artery gives off branches supplying the shoulder, thoracic wall, and scapula. It then continues as the brachial artery, which furnishes branches to the arm and elbow. Minor variations in these branches are common. However, dissection of a formalin-fixed right upper limb revealed an axillary artery that bifurcated into a superficial brachial artery, which did not give off any branches, and a posterior trunk, that gave rise to all the branches typically originating from the third part of the axillary artery and the brachial artery, including the subscapular artery, the humeral circumflex arteries, the deep brachial artery, and the collateral ulnar arteries. This rare vascular arrangement has not been previously described in Brazil. Awareness of these variations may prevent diagnostic errors and injuries during surgeries.

## Resumo

O conhecimento da vascularização é essencial para o planejamento cirúrgico e a realização de procedimentos intervencionistas na região axilar. A artéria axilar origina ramos que suprem o ombro, a parede torácica e a escápula, continuando-se então como artéria braquial, que fornece ramos para o braço e o cotovelo. Pequenas variações nesses ramos são comuns. No entanto, a dissecção de um membro superior direito fixado em formol revelou uma artéria axilar que se bifurcava em uma artéria braquial superficial, sem ramos, e em um tronco posterior, responsável por todos os ramos que tipicamente se originam da terceira porção da artéria axilar e da artéria braquial, incluindo a artéria subescapular, as artérias circunflexas umerais, a artéria braquial profunda e as artérias colaterais ulnares. Esse arranjo vascular raro não havia sido descrito anteriormente na literatura brasileira. O conhecimento dessas variações pode prevenir erros diagnósticos e lesões durante cirurgias.

## INTRODUCTION

The human arm and shoulder are primarily supplied by collateral branches of the third part of the axillary artery (AA) and the brachial artery (BA). The third part of the AA, extending from the lower border of the pectoral muscle to the lower border of the teres major muscle, gives rise to the subscapular artery. Slightly distal, the anterior and posterior circumflex arteries of the humerus commonly arise independently and encircle the anatomical neck of the humerus. The BA continues from the third part of the AA and its major collateral branches are the deep brachial artery and the superior and inferior ulnar collateral arteries.^1^

Variations in the branching patterns of the AA and BA are widely reported, with estimated prevalence ranging from 12% to 77%.^2-5^ Another variation is the presence of a superficial brachial artery (SBA), a BA that runs superficially to the median nerve.^6^

This study describes a rare anatomical variation in which the third part of the AA bifurcated into the SBA and a common trunk, which gave rise to all of the branches typically derived from the third part of the AA and the BA, resulting in an SBA lacking significant collateral branches.

## CASE REPORT

During a dissection practical using an isolated formalin-fixed right upper limb from the Laboratory of Human Anatomy at the Universidade Federal do Rio Grande do Norte, we found a rare case of a BA devoid of major collateral branches.

This study was approved by the Brazilian Human Research Ethics Committee (N^o^7.074.706; CAAE 81505524.7.0000.5537).

**Figure 1:**
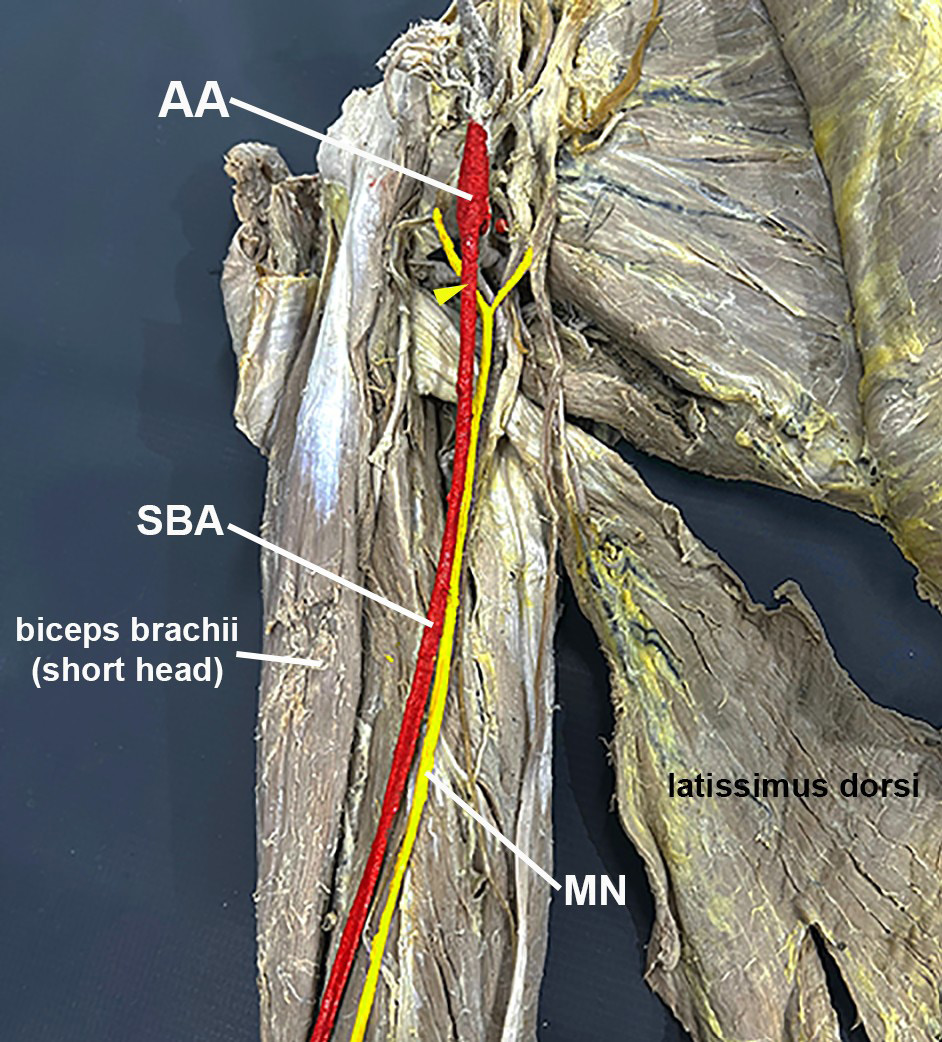
Photo of the anteromedial view of the arm and axillary area. The axillary (AA) and superficial brachial (SBA) arteries were painted red, while the median nerve (MN) and its roots were painted yellow. Note the anterior trunk of the AA passing over the lateral root of the MN (yellow arrowhead) and becoming the SBA.

A more careful dissection revealed that the AA's first and second parts were normal. However, the third part of the AA bifurcated into two main trunks right above the formation of the median nerve. The anterior trunk passed over the lateral root of the median nerve and became an SBA. This SBA ran superficially to the median nerve and medially to the biceps brachii muscle (Figure 1). It did not give rise to any considerable collateral branches at any point along its course in the arm and terminated at the cubital fossa, bifurcating into the radial and ulnar arteries.

**Figure 2:**
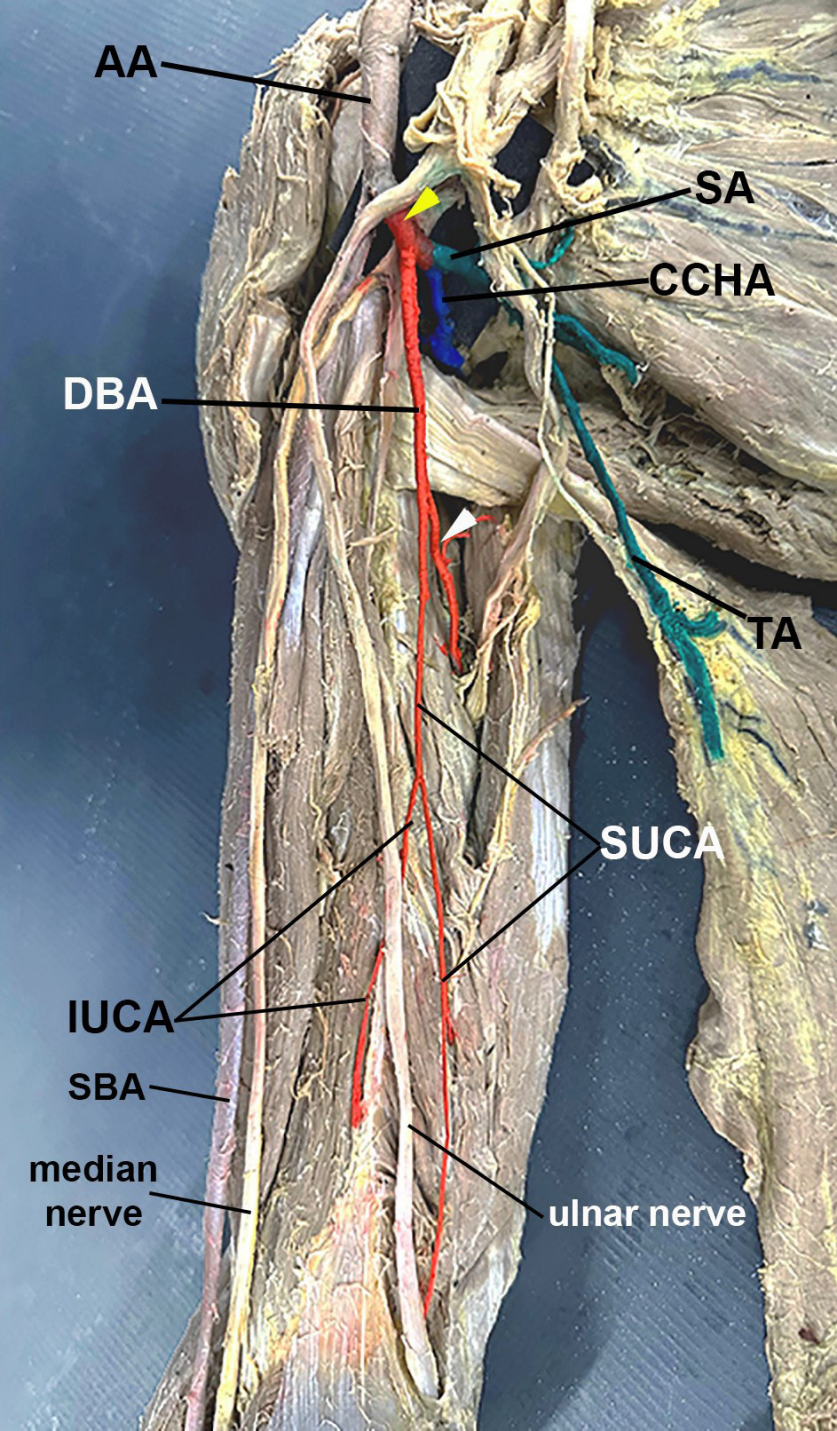
Photo of the anteromedial view of the arm. The posterior trunk was painted red (yellow arrowhead), and so were the deep brachial artery (DBA) and its branches. Note the DBA bifurcating into a branch that gives off the radial and medial collateral arteries (white arrowhead), and the superior ulnar collateral artery (SUCA). The inferior ulnar collateral artery originates from the SUCA. The common circumflex humeral artery (CCHA) was painted blue, while the subscapular artery (SA) and its branches were painted green. Note the thoracodorsal artery (TA). Other abbreviations: AA, axillary artery; SBA, superficial brachial artery.

**Figure 3:**
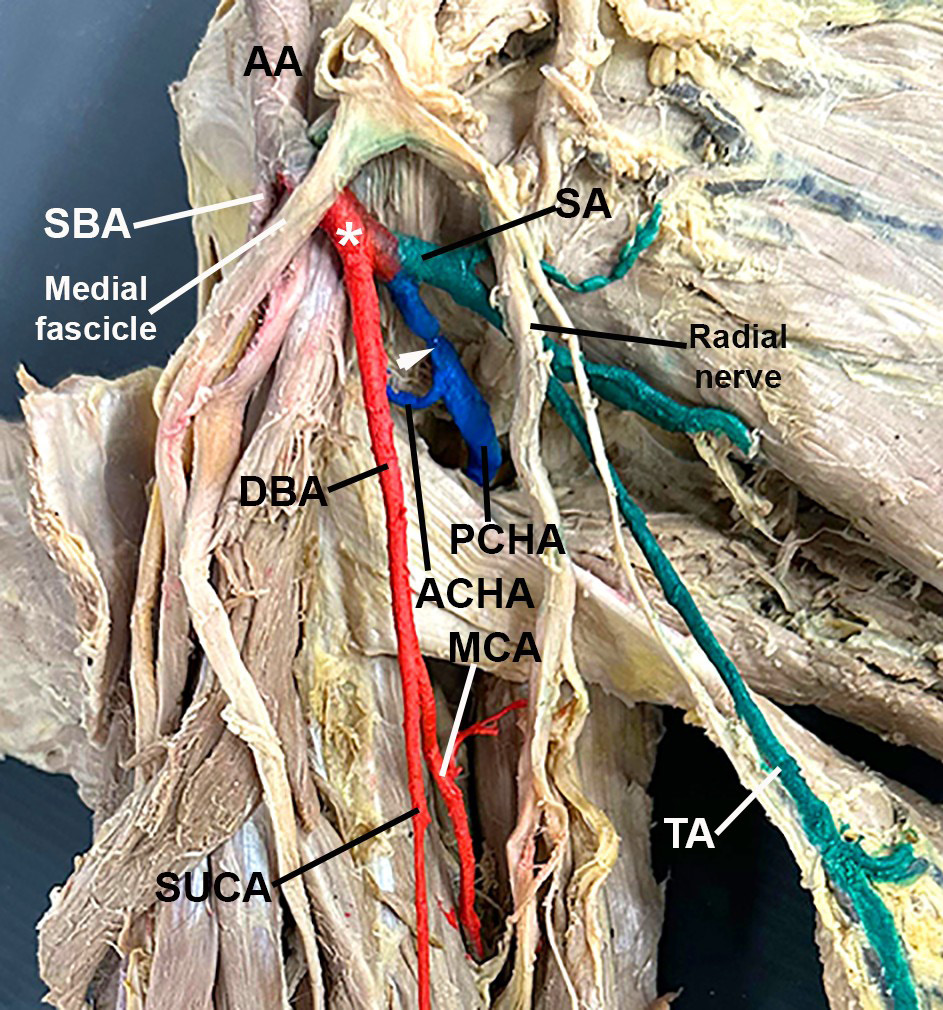
A closer view of the axillary area. The posterior trunk was painted red (white asterisk), and so was the deep brachial artery (DBA) and its branches. Note the superior ulnar collateral artery (SUCA) and the middle collateral artery (MCA). The common circumflex humeral artery (white arrowhead) was painted blue. It gives off the anterior circumflex humeral artery (ACHA) and the posterior circumflex humeral artery (PCHA). Note that the PCHA is thicker than the ACHA. The subscapular artery (SA) and its branches were painted green. Other abbreviations: AA, axillary artery; SBA, superficial brachial artery; TA, thoracodorsal artery.

The posterior trunk originated from the posterolateral aspect of the AA and, after 1.51 cm, trifurcated into a “common circumflex humeral artery”, the subscapular artery, and the deep brachial artery (Figures 2 and 3). The “common circumflex humeral artery” traveled posterolaterally and, after 1.26 cm, bifurcated into anterior and posterior circumflex humeral arteries (Figure 3). The anterior circumflex humeral artery was thin and passed deep to the coracobrachialis and biceps brachii muscles. In contrast, the thick posterior circumflex humeral artery traversed the quadrangular space alongside the axillary nerve (Figure 3). The subscapular artery arose posteromedially, giving off the “upper subscapular artery” (which supplies the subscapular muscle medially).^3^ The subscapular artery traveled for 1.71 cm before trifurcating into the circumflex scapular artery (which entered the triangular space posteriorly), the thoracodorsal artery (which accompanied the thoracodorsal nerve to the latissimus dorsi muscle inferiorly), and an artery to the teres major muscle (Figures 2 and 3). Finally, the deep brachial artery arose inferiorly from the posterior trunk (Figures 2 and 3). It traveled alongside the radial nerve for 4.68 cm before bifurcating into a branch that passed through the triangular interval, penetrated the triceps brachii muscle, and subsequently divided into the middle and radial collateral arteries, as typically described. The other branch of the deep brachial artery gave rise to the superior ulnar collateral artery (Figure 2). This artery lay anterior to the triceps brachii muscle and accompanied the ulnar nerve for 6.6 cm before giving off the inferior ulnar collateral arteries (Figure 2). The superior ulnar collateral artery passed posterior to the medial epicondyle of the humerus, while the inferior ulnar collateral artery passed anterior to it (Figure 2).

## DISCUSSION

Here, we describe for the first time in a Brazilian cadaver what seems to be an example of AA branching pattern G, as described by de Garis and Swartley in 1928. They characterized this pattern by the presence of a short common trunk originating the subscapular artery and a distally directed branch that gives rise to the common circumflex humeral artery, the deep brachial artery, and the superior ulnar collateral artery. We note that the description of pattern G does not mention the origin of the inferior ulnar collateral artery, but here we describe it as arising from the superior ulnar collateral artery. De Garis and Swartley (1928) investigated more than 500 upper limbs and found this variation in 3.6% of their sample.^7^ However, since then, pattern G variations have only been described in a few case reports, showing how rare they are.

This pattern was also associated with the presence of an SBA,^7^ which was also true in the present case. Literature indicates that the prevalence of SBAs ranges from 0.1% to 40%.^2,8,9^ Whilst not an absolute rule, it is common for the SBA to pass over the median nerve roots,^10-12^ as observed in the current case. Yang et al.^12^ classified the SBA into three types. The SBA described here corresponds to their type Ia. Although the SBA described here is divided into radial and ulnar arteries in the cubital fossa, it did not give off any major collateral branches along its trajectory. This variation seems to be more common in the types of SBA accompanied by a BA,^12,13^ which was not the case here.

Our study observed that all collateral branches typically emitted by the third portion of the AA and the BA originated from the posterior trunk. The formation of arterial trunks in the third part of the AA is a common anatomical variation, with prevalence ranging from 6% to 74.6%.^4,5,14,15^ However, the specific vessels that constitute these trunks can vary. Small trunks are more common, which means two or three arteries, such as the posterior and anterior circumflex humeral, subscapular, or deep brachial arteries, arising together.^16-21^ Nonetheless, a common trunk that gives rise to all the branches of the third portion of the AA and BA is very rare. Soubhagya et al.^22^ and Vijaya et al.^23^ described trunks that gave rise to the arteries from the third part of the AA and the BA, except the inferior collateral ulnar artery. Aastha et al.^24^ and Rao et al.^25^ described cases similar to ours, but no “common circumflex humeral artery” was formed in either of their cases. All of these examples are variations of pattern G, in common with the case reported here, but with slight differences, which make the present case unique.

The superficial position of the SBA makes it more vulnerable to trauma, and it may be misinterpreted as a vein. It may also be the cause of median nerve entrapment neuropathy. Familiarity with variations in the vascular anatomy of the shoulder and arm is essential for accurate interpretation of angiographic findings and effective surgical planning. It may prevent diagnostic errors and influence interventional surgical procedures, including breast cancer surgery or axillary cavity exploration. Therefore, remarkable variations such as those described in this study hold practical significance for professionals in the fields of anatomy, radiology, anesthesiology, and plastic and vascular surgery.

## Data Availability

The data supporting this study are available upon request to the corresponding author, JCC, due to departmental rule.
